# Aggregates, Crystals, Gels, and Amyloids: Intracellular and Extracellular Phenotypes at the Crossroads of Immunoglobulin Physicochemical Property and Cell Physiology

**DOI:** 10.1155/2013/604867

**Published:** 2013-03-05

**Authors:** Haruki Hasegawa

**Affiliations:** Department of Therapeutic Discovery, Amgen Inc., 1201 Amgen Court West, Seattle, WA 98119, USA

## Abstract

Recombinant immunoglobulins comprise an important class of human therapeutics. Although specific immunoglobulins can be purposefully raised against desired antigen targets by various methods, identifying an immunoglobulin clone that simultaneously possesses potent therapeutic activities and desirable manufacturing-related attributes often turns out to be challenging. The variable domains of individual immunoglobulins primarily define the unique antigen specificities and binding affinities inherent to each clone. The primary sequence of the variable domains also specifies the unique physicochemical properties that modulate various aspects of individual immunoglobulin life cycle, starting from the biosynthetic steps in the endoplasmic reticulum, secretory pathway trafficking, secretion, and the fate in the extracellular space and in the endosome-lysosome system. Because of the diverse repertoire of immunoglobulin physicochemical properties, some immunoglobulin clones' intrinsic properties may manifest as intriguing cellular phenotypes, unusual solution behaviors, and serious pathologic outcomes that are of scientific and clinical importance. To gain renewed insights into identifying manufacturable therapeutic antibodies, this paper catalogs important intracellular and extracellular phenotypes induced by various subsets of immunoglobulin clones occupying different niches of diverse physicochemical repertoire space. Both intrinsic and extrinsic factors that make certain immunoglobulin clones desirable or undesirable for large-scale manufacturing and therapeutic use are summarized.

## 1. Introduction

Immunoglobulins (Igs) are the important mediators of humoral immunity. Because of their variable domain primary sequence diversity that is somatically generated via combinatorial gene segment joining of germline-encoded DNA and hypermutations [1], the repertoire of Ig clones is estimated to exceed 10^10^ [2]. Such sequence diversity is advantageous when it comes to conferring comprehensive foreign antigen coverage to protect the host, but the same sequence variations—in conjunction with a structural constraint of a scaffold-based protein design—can pose a significant biosynthetic challenge to produce and secrete each Ig clone in an equally efficient manner. Because of the sequence diversity within the variable domains, individual Igs possess different physicochemical attributes unique to each clone that may impose differential resource load to the cell and thus may modulate various biosynthetic processes, including the rate of protein synthesis, folding kinetics, assembly efficiency, interactions with ER quality control components, and intracellular trafficking. Similarly, because of the immense collection of physicochemical properties, distinct clones of Ig would certainly behave differently after secreted to the extracellular space. The guiding theme of this paper is to highlight the roles of variable domain sequences in influencing various aspects of Ig life cycle as a macromolecule and is to illustrate the challenges of identifying Ig clones suitable for large-scale manufacturing and for therapeutic use. The information reviewed in this paper is useful in guiding lead candidate selection and optimization strategies as well as in aiding protein and cell phenotype engineering to accelerate therapeutic antibody discovery.

## 2. Russell Body Biogenesis: A Cellular Response to Biosynthetically Challenging Immunoglobulin Clones

### 2.1. Historical Perspectives on Russell Body Phenotype: Mott Cells, Morular Cells, and Grape Cells

Aberrant intracellular globules in lymphocytes and plasma cells have captivated cell biologists for more than a century. The globular cytoplasmic structure called Russell body (RB) was named after William Russell, a pathologist who first reported such spherules and interpreted them as intracellular parasitic fungi, which he regarded as an etiological cause of cancer [3, 4]. The terms such as Russell's fuchsin bodies, Russell's bodies, and Russell bodies of plasma cells had already been widely used by the 1920s [5–7] even when the origin of plasma cells was still actively debated. Since then, RBs have been extensively characterized morphologically by various methods including periodic acid-Schiff staining [8, 9] and electron microscopy [10, 11]. After a lengthy controversy (discussed in [6, 12]) on the origin and the composition of those globular subcellular structures, it was concluded eventually that RBs were composed of condensed Igs and were surrounded by the membranes of rough endoplasmic reticulum (ER) [8, 13–19]. In spite of Russell's original assertion, RBs are now defined as intracellular inclusions of aggregated Igs enclosed in dilated ER; the biogenesis of which takes place when there is an imbalance between Ig synthesis and the combined rates of folding and degradation [20]. 

Plasma cells and lymphocytes containing RBs came to be called Mott cells based on the work by Mott [21] that described the presence of morular cells in Trypanosoma-infected animal brains. It turned out that morular cells were in fact plasma cells housing Ig inclusions composed largely of IgMs [22–24]. Mott cells have been detected abundantly in lymphoid organs of mice and humans with autoimmune diseases [25–27] and also in other pathologic conditions such as leukemias, multiple myelomas, monoclonal gammopathies, and chronic infections [28–31]. Importantly, it was also noted very early that the presence of Mott cells was not always associated with obvious pathological processes [8, 14, 30, 32].

RB-containing plasma cells were also called grape cells at one point. Grape cells were described in the bone marrow of multiple myeloma patients [33] and also in the patients associated with hyperglobulinemia and plasma cell hyperplasia [34]. Grape cells were also experimentally inducible in the spleen of hyperimmunized rabbits [35]. There was an earnest attempt to differentiate the grape cells from RB-containing Mott cells by subtle staining patterns and color differences, but “on morphologic grounds there seems to be no difference” [34].

While the case reports on RB morphology accrued, the understanding of their biogenesis had remained largely unclear for a long period. One of the earliest views on the origin of RB-housing cells was that they are the normal end stage of plasma cells undergoing degeneration after having fulfilled the secretory functions [36, 37]. Based on the apparent correlation between Mott cell frequency in avian lymph tissues and the bird age, Wight et al. [38] proposed that its occurrence was a sign of plasma cell senescence. Because Mott cells also occurred in normal spleens during mitogenic stimulation, Nowak et al. [39] discussed that Mott cells were a normal differentiation state of the B-cell lineage, but were caused by uncontrolled production of abnormal Igs. Similarly, a demonstration of Mott cell differentiation from an expanded clone of B lymphocytes *in vitro* apparently supported the view that RB formation was part of normal B-cell differentiation processes [29]. There was also an attempt to identify a gene that increases the susceptibility of B cells to form RB in mice [40]. Although the authors claimed to have mapped the locus to chromosome 4, near a gene cluster of TNFR family, the identity of such gene, let alone its role in RB biogenesis, has not been elucidated to date. While many investigators focused on the contributions of cellular physiology in RB formation, White [14] already had the foresights to hypothesize the roles of varying physical properties of individual antibodies in RB formation where some clones of antibodies are more easily precipitated within the cytoplasm of plasma cells than others.

### 2.2. Hybridoma Cell Lines as a Model System to Study RB Biogenesis

The establishment of hybridoma cell lines that spontaneously develop RBs enabled a detailed cellular characterization and biochemical analysis on their compositions *in vitro*. For example, a hybridoma clone A2-3.88, that expresses a clone of *λ*LC without detectable HCs, did not secrete *λ*LCs to the culture medium in spite of the protein synthesis. Instead, nearly all the cells exhibited Mott cell phenotype. Despite the phenotype, cell growth rate was comparable to that of normal hybridomas; the Mott cells did undergo cell division while harboring RBs in their cytoplasm [41]. Alanen et al. [42, 43] characterized a different panel of Mott cell hybridoma clones which all synthesizes both HC and LC subunits. Although HCs and LCs appeared to assemble in the ER, the formed IgGs were retained in this compartment without getting secreted. Because endogenously expressed cell surface resident proteins were delivered correctly to the plasma membrane, a generalized secretory system failure was ruled out from the possible causes of RB induction in this hybridoma.

Mice homozygous for “viable motheaten (me^v^)” allele show chronic polyclonal B-cell activation and develop severe autoimmune as well as immunodeficiency phenotypes [44]. During the investigation for homozygosity for me^v^ mutation, Shultz et al. found that the mutant mice abundantly accumulated IgM-positive Mott type plasma cells in lymph nodes and spleen after five weeks of age [26]. If the thymus was removed neonatally, the occurrence of large number of Mott cells in lymphoid tissues was prevented, suggesting an obligate role of functional T cells for the development of Mott cell phenotype *in vivo* [26]. Although the mechanisms of abundant Mott cell generation were not fully elucidated, Shultz and co-workers hypothesized that B cells are spontaneously activated to proliferate and become plasma cells irrespective of the specificity of surface displayed B-cell receptor, thus resulting in uncontrolled Ig production [26, 27, 45]. By taking advantage of the abundant source of Mott cells and to characterize RB biogenesis *in vitro*, cells from cervical lymph nodes or spleen of me^v^/me^v^ mice were fused to a nonsecreting SP2/0 myeloma to establish a panel of IgM expressing hybridoma lines that retained the characteristics of Mott cells in high frequency, roughly one-third of hybridomas generated [27]. In those Mott cell hybridomas, IgM accumulated rapidly as insoluble intracellular aggregates which cannot be secreted or degraded, thereby giving the polypeptides 10 times longer intracellular half-life than normal [27]. By using an IgG1-secreting myeloma P3X63Ag-8 as an additional fusion partner, Schweitzer et al. showed that RBs were composed of IgM (me^v^/me^v^ mice origin), but the fusion partner-derived IgG1 did not accumulate in the RBs and was secreted normally from the same hybridoma [27]. Protein aggregation leading to RB formation thus occurred selectively to the IgM clone, but not to the IgG1 clone expressed simultaneously in the same hybridoma cell. Again, the results suggested that RB phenotype occurred not because of the secretory defects of the expressing cell hosts, but rather such propensity was intrinsic to some Ig clones [27].

Hybridoma technologies are still widely used in biotechnology to search for therapeutic antibody candidates. To generate intact human antibodies with fully human variable domains, various transgenic mice harboring human immunoglobulin loci have been developed [46]. Despite the popularity of hybridoma-based antibody discovery methods which often rely on multiple immunization schedules, the frequency of Mott cell induction in immunized mice and in established hybridoma, let alone the phenotypic stability of the latter, during a typical antibody discovery campaign, has not been reported to date. Such investigation will be essential in understanding the biosynthetic efficiency differences, if any, between the endogenous expression in the homologous cellular systems of hybridoma and the recombinant expression in heterologous cell hosts (e.g., HEK 293 and CHO) using an identical set of IgG clones.

### 2.3. Constant Domain Deletion Mutants and Russell Body Biogenesis

The first constant domain of the HC subunit (CH1) plays important roles in regulating Ig biosynthesis including the retention of free HCs by the BiP-mediated ER quality control mechanisms and the covalent assembly with the LC subunits. Despite such critical importance, the CH1 domain deletion can occur naturally via errors in gene recombination [47, 48]. The deletion of CH1 domain is believed to circumvent the toxicity of full-length HC protein expression in the absence of LC synthesis in homologous systems [49]. The ΔCH1 mutation is also known to facilitate HC-only antibody secretion by bypassing BiP-mediated retention in the ER [50]. In camelids, for instance, considerable amounts of HC only dimers lacking the CH1 domain are secreted to the serum as functional HC antibodies [51, 52]. In a heterogeneous group of human HC diseases (see below), truncated HCs lacking the CH1 domain region are secreted from the cells and often deposited to various tissues to cause structural and functional damages [53–55]. What then is the role of CH1 domain in RB formation? Given that some Mott cell hybridomas did express full-length HCs and LCs [27, 56], the CH1 deletion was not a prerequisite for RB biogenesis. In a study using knock-in mice, Corcos et al. presented evidence that internally truncated HC alone was not sufficient to induce RB formation *in vivo* either [57]. Therefore, *cis*-acting determinants that contribute to induce RB formation are located elsewhere on the HC polypeptides.

Although the constant domain deletion by itself is not a critical inducer of RB biogenesis, many studies in the past employed various isotypes of recombinant ΔCH1 HCs as model systems. Valetti et al. [58] showed that Mott cell phenotypes arise when the Cys residue located within the secretory tail piece at the C-terminus of ΔCH1 *μ* chain and the LC formed insoluble lattice that resists efficient degradation. The RB formation was proposed as a general cellular response to an accumulation of insoluble protein aggregates in the ER [58]. Mattioli et al. also showed that the recombinant expression of ΔCH1 *μ* chain alone in NS0 cell (a mouse myeloma that synthesizes neither HC nor LC) does not normally induce RB formation; because in the absence of LCs, the ΔCH1 *μ* chain cannot form aggregates. However, by manipulating the rate of protein synthesis and degradation pharmacologically, they demonstrated that ΔCH1 *μ* chain expression alone can induce RBs in NS0 cell [59]. This was the first experimental demonstration where the imbalance between protein synthesis and degradation can lead to RB formation even when the protein itself was innocuous. Kaloff and Haas [60] reported that when J558L cell (a mouse myeloma that synthesizes and secretes endogenous *λ*LC, but no HC) was transfected with *γ*3-HC either in full-length or ΔCH1 context, RBs formed only when the ΔCH1 mutant was expressed although ΔCH1 HCs and the endogenously expressed *λ*LCs do not assemble covalently. Despite the apparent Mott cell phenotype, the transfected J558L myeloma secreted a noncovalently assembled IgG3, implying that the RB formation may not always be an all-or-nothing event when it comes to Ig secretion [60].

The system of ER chaperones and folding enzymes is an integral part of Ig subunit chain folding and assembly reactions [61, 62]. Although many ER resident chaperones regulate Ig assembly by interacting with CH1 domain [63], some aspects of the roles of ER quality control mechanisms during RB biogenesis were investigated using a ΔCH1 *μ* chain. By overexpressing or silencing key components of the ER quality control system, Ronzoni et al. showed that increased level of HRD1 and ERdj5 (both functions in the ER-associated degradation steps, or ERAD) inhibited RB formation of the ΔCH1 *μ* chain in HeLa cells [64]. In contrast, an overexpression of ERp44 (a distal component) exacerbated, while an overexpression of PDI (a proximal component) ameliorated, the insoluble aggregate formation of the model ΔCH1 *μ* chain [64]. Interestingly, both overexpression and silencing of Ero1*α* prevented the RB formation, potentially indicating a role for the strict redox balance in the ΔCH1 *μ* chain RB formation [64]. Because of the presence of secretory tail piece that promotes polymerization of IgMs, the biosynthetic complexity of IgMs could be quite distinct from that of IgGs. Whether those observations are restricted to a certain clone of mutant ΔCH1 *μ* chains or have a far-reaching general impact on structurally normal Igs of different isotypes have yet to be determined.

Lastly, Corcos et al. [57] compared the RB forming frequency of plasma cells derived from knock-in animals (*μ*NR mice) expressing truncated *μ* chain lacking CH1-2 domains [65] with that of plasma cells derived from *μ*NRL^−/−^ mice which are devoid of LC expression in addition to the CH1-2 deletion [66]. The study showed that truncated *μ*-HC frequently induced RB formation in the LC null background of *μ*NRL^−/−^ mice, but the RB formation was largely suppressed when the LCs were present in the *μ*NR mice background. The results clearly suggested an important protective role of LCs in preventing the truncated HCs from aggregating into RBs *in vivo*. The LC deficiency can therefore lead to RB phenotypes in a certain genetic background.

### 2.4. Roles of the Variable Domain Sequences in Russell Body Formation

The potential importance of variable domain sequences in determining the RB propensity was first discussed in 1992 by two groups [56, 67]. Tarlinton et al. [56] showed that two clonally unrelated IgM Mott cell hybridomas utilized unmutated germline V gene segments for both *μ*HC and *κ*LC and there were no mutations in the constant regions; therefore, no structural abnormalities or even somatic hypermutations were necessary to induce RBs. In a series of HC-LC mix and match coexpression experiments using selected IgM clones, the Mott phenotype occurred only when the autologous (or cognate) HC-LC pair was coexpressed. Because the RB phenotype was dependent on a particular combination of VH and VL domains, Tarlinton et al. [56] proposed that the antigen specificity created by this VH/VL pair had caused the phenotype—perhaps by reacting to itself or some unknown intra-ER components in a way to prevent correct folding and assembly. Importantly, however, an isotype switching from IgM to IgG1 abrogated RB formation although the VH and VL were identical. A very similar result was reported for a different IgM producing Mott cell hybridoma NYCH. A set of subunit chain swapping experiments again suggested that RBs were induced only when the autologous *κ*LC and *μ*HC were coexpressed [67]. Similarly, when the HC isotype was switched from *μ* to either *γ*1 or *γ*3, the RB propensity disappeared [67]. Both groups interpreted that it was the antigen specificity generated by the VH/VL pair that was important for RB biogenesis. In retrospect, however, the same set of observations can be explained differently. Namely, it may actually be the particular physicochemical properties generated by the autologous VH/VL pair (not necessarily the antigen specificity *per se*) that induced RB formation. These findings additionally implicated an important role of HC isotypes in modulating the RB-inducing threshold. In conclusion, the two independent studies laid the foundation to understand the mechanisms of RB formation in a clone-specific and isotype-dependent manner, without postulating gross structural abnormalities.

### 2.5. Interplay between the Cellular Protein Homeostasis and the Physicochemical Properties of Individual Immunoglobulin Clones

**Figure 1:**
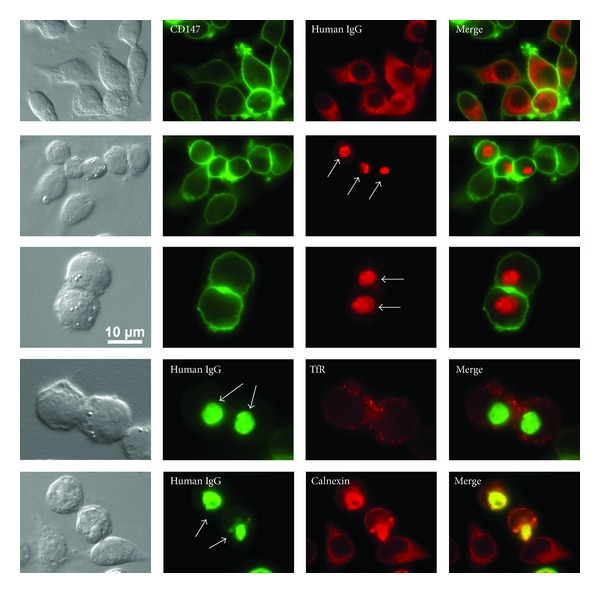
Immunofluorescent micrographs of HEK 293 cells expressing recombinant human IgGs. First three rows: cells were costained with anti-CD147 (green) and anti-human IgG (red). Fourth row: cells were costained with anti-human IgG (green) and anti-transferrin receptor (TfR) (red). Bottom row: Cells were co-stained with anti-human IgG (green) and anti-calnexin (red). Top row: the cells were transfected to express a high-secreting IgG clone. Recombinant human IgGs populated cytoplasmic reticular structures in transfected cells at steady state. Second row: cells express a low-secreting IgG clone. IgGs aggregated into Russell bodies in transfected cells. Russell bodies are pointed by arrows. Third through fifth rows: Russell body-housing cells are shown in a higher magnification. RB formation was typically accompanied by cell rounding in HEK 293 cells.

By employing heterologous cell hosts such as HEK 293 or CHO and a robust protein production platform, Stoops et al. [68] recently characterized the clonal differences in RB formation using a panel of functionally selected recombinant human IgG clones. Although all the tested model IgG clones were structurally normal, individual clones exhibited markedly different thresholds to result in RB phenotypes under normal cell culture conditions. For example, highly secreted IgG clones (~100–300 mg/L titers) had very low (or no) propensity to induce RB formation, whereas poorly secreted IgG clones (~1–20 mg/L) resulted in RB phenotypes in the majority of Ig expressing cells (see Figure 1). In good agreement with the RB phenotype (or lack thereof), the intracellular pool of high-secreting IgG clones remained largely soluble, while the poorly secreting IgG clones became insoluble aggregates and were not readily secreted or degraded as suggested by an extremely long intracellular residence time, which indirectly indicated that RB-prone IgG clones are poor substrates for ERAD machinery and are not amenable for vesicular packaging at the ER exit site due to insoluble aggregate formation. Stoops et al. [68] also reported that, unlike the homologous models of Mott cell hybridomas, the health of heterologous cell hosts deteriorated faster when the cells express RB-inducing IgG clones. Because the studies were carried out using IgG clones sharing identical constant domains, it was most likely that the apparent differences in secretion titer, RB-inducing propensity, effects on cell viability were all influenced by the intrinsic properties embedded in the variable domain sequences that define individual IgG clones.

**Figure 2:**
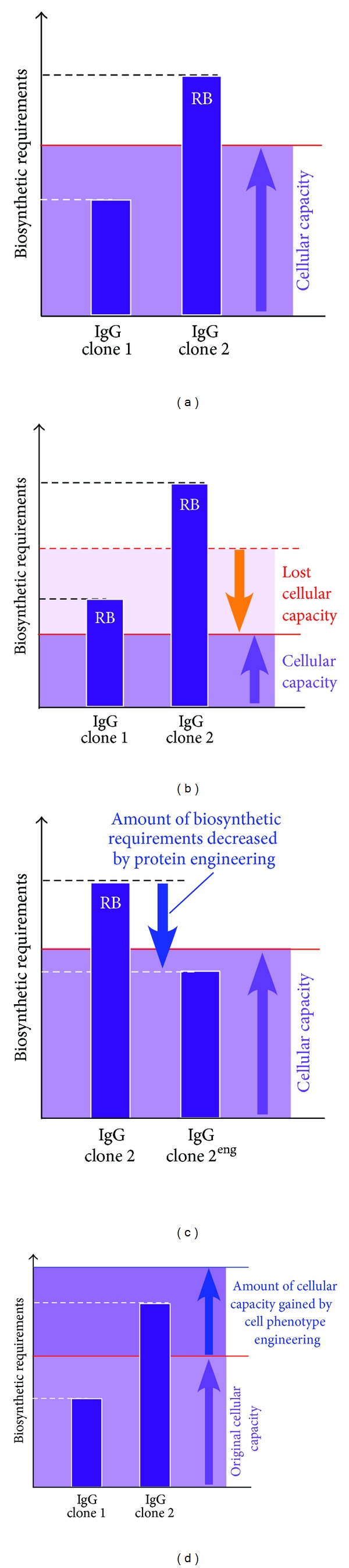
Schematic representation of the relationship between individual IgG clones' biosynthetic requirements and intrinsic cellular capacity. (a) In this hypothetical setting, two IgG clones 1 and 2 have different levels of resource requirements during biosynthesis (shown in the vertical axis). The cells have sufficient capacity to support clone 1's biosynthesis, but do not have the capacity to meet the requirement of clone 2. Clone 2 ends up aggregating into Russell body (RB). (b) The same pair of clones were expressed in the same cell background. In this hypothetical setting, however, the cells have lost a part of their capacity due to stress, senescence, pathological changes, and so forth. As a result, both IgG clones come to develop RB phenotypes. (c) An example of successful protein engineering that reduced the biosynthetic requirements of IgG clone 2. Engineered clone 2^eng^ no longer induces RB in the same cell host. (d) In this setting, cell phenotype engineering strategies increased the overall capacity of the cell host. The clone 2 no longer forms RB in the engineered cell host.

The intrinsic properties genetically encoded in the variable domain sequences predisposed some IgG clones to be more susceptible to aggregation during biosynthesis, but the extrinsic factors also played critical roles in RB biogenesis. Using high-secreting, innocuous IgG clones that normally do not induce RBs, Stoops et al. [68] showed that stressful cell culture conditions (i.e., heat shock or thapsigargin treatment) that disrupt cellular protein homeostasis can unmask aggregation propensities from these IgG clones by compromising cellular capacities. However, the frequency of RB formation under different adverse cell culture conditions still varied from clone to clone, again, indicating that each IgG clone has a unique inherent threshold to induce RB formation even under abnormal cell growth conditions. In summary, this work suggested that if the physicochemical properties are identical, differences in the cellular context or cellular capacity dictate how efficiently the IgG biosynthesis and secretion can take place. Therefore, environmental stress or infectious episodes that may compromise ER capacity or general cellular functions can precipitously induce protein aggregation and elevate the prevalence of RB formation (Figure 2).

### 2.6. Dissecting the Roles of HC and LC in RB Formation

In homologous experimental systems, a sustained expression of full-length HCs in the absence of LC synthesis turns out to be difficult because of the proteotoxicity that eradicates such cell population, combined with the cellular mechanisms that delete CH1 domain to circumvent the toxicity [49]. Accordingly, the precise roles of full-length HCs in RB formation have not been effectively addressed. In contrast, the presence of LC-only Mott cell hybridoma [41] has implicated that some LC clones can induce RBs by themselves in the absence of HCs although the Mott phenotype was somewhat unstable due to the spontaneous loss of LC synthesis in this hybridoma cell line. Whether all the LCs equally have such RB-inducing propensities remained largely unknown until recently.

By taking advantage of a robust recombinant expression system, Stoops et al. [68] assessed the contribution of each subunit chain in RB biogenesis by expressing individual subunit chains separately. When individually expressed, all seven full-length HCs derived from seven independent IgG clones were highly prone to induce RBs and were barely secreted to the culture media. Intact full-length IgG-HCs therefore tend to aggregate into RBs in the absence of LCs. In contrast, the propensity of individual LCs to induce RBs as well as to be secreted by themselves showed significant clonal variations, apparently depending on whether the LCs were derived from high-secreting or low-secreting IgG clones [68]. LCs derived from high-secreting clones rarely induced detectable RBs and were efficiently secreted to the extracellular space as monomers and dimers. The LCs derived from poorly secreting IgG clones, on the other hand, induced RBs in nearly all the expressing cells and very poorly secreted by themselves. These results suggested that not all the LCs are equally competent in their ability to reach secretion-competent conformation by themselves, and also in their ability to facilitate HC-LC assembly as a chaperone or as a structural component. In other words, depending on the level of intrinsic competency, some LCs are more capable of assisting Ig assembly and preventing HCs from aggregating into RBs. Because the same isotype LCs can show significant differences, the “competency” of the individual LCs appeared to be encoded in the variable domain sequences of individual LC clones. Furthermore, by carrying out a panel of HC-LC mix and match coexpression experiments, Stoops et al. [68] demonstrated that the LC's high competency was necessary, but not sufficient to ensure the efficient biosynthesis and high level secretion. When the LCs were competent enough, the efficiency of IgG assembly and secretion appeared to be limited by the compatibility between the VH and VL sequences of the assembling subunits. Stoops et al. [68] also showed that autologous, or cognate, HC-LC pairs do not always ensure maximum biosynthetic efficiency; which importantly underscored the lack of selective pressure and mechanisms in fitting the “right” VH/VL pairs to maximize the secretory productivity during Ig biosynthesis. It is therefore inevitable that the efficiency of subunit assembly reactions (and the RB forming propensity) varies from clone to clone *a priori*, because of an inherent limitation to accommodate a diverse set of sequences in one scaffold-based structural configuration.

### 2.7. Implications of Differential RB-Inducing Propensities for Therapeutic Antibody Discovery Research

The differential RB-inducing properties among different Ig clones are likely to be governed by the intrinsic physicochemical properties embedded in the VH and VL domain sequences, although in some studies the HC isotypes also influenced the threshold. As demonstrated by Stoops et al. [68], both intrinsic threshold that is genetically encoded in the molecule itself and the extrinsic factors that influence intracellular protein homeostasis modulate whether a particular IgG clone will induce RB phenotype in a given cellular context under a given cell growth condition (Figure 2). The high-secreting IgG clones were clearly underpinned by favorable properties that allow those IgG clones to fold, assemble, and secrete robustly—although what statistically significant features will render some IgG clones fitter than others remains unclear.

While the mechanisms of the covalent assembly steps between CH1 and CL as well as the roles of ER chaperone system and quality control mechanisms in this process have been well characterized at the molecular level [61, 62, 69], the understanding on the roles of VH/VL domains in the assembly reaction is still insufficient. There are apparent pairing preferences in small subset of germline VH/VL variability subgroup sequences when a large number of archived Ig clones in the KabatMan database were statistically analyzed [70]. Furthermore, when tested for its binding affinity using purified components* in vitro*, some VH/VL pairs preferentially associated with higher affinities regardless of whether the VH and VL were autologous or not [71, 72]. It is currently unknown whether such differences in the non-covalent molecular interactions among different germline-encoded VH/VL pairs provide a potential mechanism to determine HC-LC compatibility. It is attractive to speculate that interface residues brought by a certain pair of variability subgroups are inherently more compatible in Ig assembly reaction (e.g., counterbalancing charges or shape differences), and less prone to aggregate into RBs during biosynthesis.

Somatic hypermutations may also play roles in VH/VL compatibility both in negative and positive way. There is in fact literature evidence illustrating the significance of hypermutations in VH/VL compatibility. By using anti-phosphocholine antibody clone T15 as a model, Rittenberg's group carried out a mutagenesis to isolate four HC variants with mutations in the CDR2 that impede subunit assembly and secretion [73]. When coexpressed with its cognate LC subunit, the secretion was 90% less than that of the parental HC-LC pair. Because somatic hypermutations can take place in both subunits independently, when similar mutagenesis was carried out on the LC's variable domain sequence, a LC variant with S16G/K17E mutation was able to restore the assembly and the secretion when coexpressed with the previously isolated secretion-impairing HC mutants [74]. It was not reported whether the nonsecretory pairs gave rise to Mott cell phenotypes in their study. However, it was an important example where deleterious secretion-impairing mutations on one variable domain can be overcome by compensatory mutations in the variable domain of the other subunit. Various groups have reported different point mutations that abolish efficient Ig assembly and secretion to date [75–78]. It remains to be experimentally shown, but still is an attractive possibility to consider that somatic hypermutations are able to restore those secretion defects by introducing compensatory mutations on the other subunit.

Given the diversity of primary sequences and the physicochemical property repertoire, it is likely that not all the IgG clones may be suitable for the cell-based overexpression strategies being used in biotech industry today. Therefore, when it comes to selecting the lead candidates, it is beneficial to assess their overexpression fitness in addition to focusing on identifying antibody clones with potent biological functions. RB biogenesis has been a subject of basic molecular cell biology research, but recent studies have projected hitherto unrecognized potential values of RBs in the biotechnology of recombinant IgG expression. For example, Stoops et al. [68] proposed that the differential RB-inducing propensity can be a useful criterion to differentiate IgG clones that are suited for overexpression strategy from those that are not. RB phenotype screening assay thus can potentially help mitigate the risk of unknowingly selecting and advancing IgG clones that later turn out to be unsuitable for large-scale manufacturing. Similarly, monitoring the RB-inducing propensity may guide antibody engineering strategies to assess the benefit and the cost of particular residue substitution on the biosynthetic efficiency (Figure 2). Likewise, such assay might be useful as a rational tool to prioritize synthetic antibodies generated by various display technologies by rank-ordering their overexpression fitness in mammalian cell system. Furthermore, adaptation to an automated image-based screening methodology may dramatically increase the throughput of phenotype screening if a large number of IgG clones need to be examined efficiently. Despite those intriguing potentials, the true value of RB phenotype screening has not been fully explored.

## 3. Crystalline Body Biogenesis: Mechanisms of Intracellular Immunoglobulin Crystal Formation

### 3.1. ER Storage: Crystallization at the Site of Immunoglobulin Synthesis

Since the first report on crystalline bodies (CBs) in 1917 by Glaus [79], intracellular crystals associated with plasma cells and lymphocytes have been documented from a variety of human pathological sources such as plasmacytoma, myeloma, and chronic lymphocytic leukemia as well as non-Hodgkin's lymphoma [80]. The shapes of the CBs vary significantly from a rod-shape, rod with tapering ends, rhombohedric, cubic, to oval and round, reflecting that numerous Ig clones endowed with various physicochemical attributes can give rise to this group of cellular phenotypes. In some cases, crystal-housing cells were noticeably enlarged compared to the non-crystal-laden cells of the same type [81]. By various staining techniques, the crystals occurring within the cytoplasm of plasma cells were shown to be similar in composition to RBs, and the Ig nature of the crystals was suggested by the mid 20th century [14, 82, 83]. Although numerous clinical cases have been published to date, they were largely limited to survey their subcellular localization and isotypes of intracellular crystal Igs.

Similar to what was shown for RBs, CBs have been reported to develop in the lumen of the ER where Ig synthesis, folding, and assembly take place. In autoimmune me^v^/me^v^ mice (see Section 2.2), CB-housing plasma cells can be found side by side with RB-harboring Mott cells in the same lymph nodes [26]. In clinical settings, no particular HC or LC isotype was overrepresented as the components of CBs [15, 83–94]. However, there are two cases where intracellular crystals were composed of the LC subunit only; one report was on a human *λ*LC expressed in bone marrow derived B lymphocytes from chronic lymphocytic leukemia patient [95] and the other was on a murine hybridoma clone F10 expressing a *κ*LC [96]. In the latter case of mouse *κ*LC hybridoma, the crystals localized in the ER lumen by electron microscopy [97]. Because there were no primary sequence abnormalities in the constant domain of the F10 *κ*LC, the authors concluded that the crystallization propensities came from the variable domain [97].

What is then causing the intracellular Ig crystallization? Goldberg [83] was the first to speculate that intracellular crystals were composed of structurally abnormal Igs that the cells were unable to secrete. What followed this early hypothesis was a series of reports on crystallizable human Igs wtih gross structural abnormalities such as a deletion in the HC hinge region [98–100]. Because no alternative models were convincingly put forth, this abnormal protein hypothesis has been perpetuated until very recently as evidenced by a speculation stating “structurally abnormal immunoglobulins synthesized in excess may crystallize giving rise to needle-shaped or rhomboid crystals” [101]. While such view remained popular, cell biologically more fundamental questions were already asked by Zettergren [81] in 1949 as to whether the crystallization was because of the protein-producing capacity in the plasma cells or is a manifestation of resorptive property of these cells (or thesaurismosis). There are in fact two types of intracellular crystallization events for immunoglobulins. The one is in the ER (Section 3.2), and the other takes place in the endosome/lysosome compartments where phagocytosed Igs traffic for recycling to the circulation or for degradation (Section 3.3).

### 3.2. Underlying Mechanisms of IgG Crystal Formation in the ER

Unexpected new insights into the intra-ER antibody crystallization mechanisms came from a recent work, roughly a century after the first report, using CHO cells and HEK 293 cells that overexpress a recombinant human IgG clone [102]. Structural or sequence abnormalities of Igs had been emphasized as the primary cause of the CB phenotypes. However, as evident from this recent work, it was not the structural abnormalities, if any, but simply was the particular set of physicochemical properties embedded in the variable domain of the Ig clones. In fact, the IgG species that made up the intra-ER crystals in this study was not only reversibly soluble, but also correctly assembled and folded. Once allowed to become soluble again after isolated from the crystal-housing cells, the folding stability of intracellular crystal-derived IgG was equivalent to that of the secreted counterpart. Likewise, intracellular crystal-derived IgG retained antigen binding ability equivalent to the secreted IgGs which suggested the folding of the Fab domain was correct. However, in agreement with their ER storage, the IgGs comprising the intracellular crystals remained sensitive to endoglycosidase H [102]. The IgG species making up the intra-ER crystals therefore had the attributes otherwise considered to be “exportable” from the ER, but nonetheless accumulated and crystallized in the ER lumen. A structurally and functionally normal IgG can therefore meet the physicochemical criteria that result in crystallization in the ER.

In this recent example, engineered CHO cells spontaneously developed rod-shaped CBs in the ER under normal cell culture conditions, and the CB formation was typically accompanied by a marked cell enlargement and multinucleation. The phenotype implicated that intracellular crystals physically impeded cytokinesis without hampering karyokinesis and cell volume growth. Intracellular crystal growth typically continued until the crystals outgrew cell sizes to the point of cell rupturing [102]. Therefore, once this phenotype appears, the cells seem to have a finite life span depending on how fast the growing crystals can breach the membrane integrity, which is perhaps also influenced by the shapes of the CB (which in turn is determined by the physicochemical properties of the Igs).

**Figure 3:**
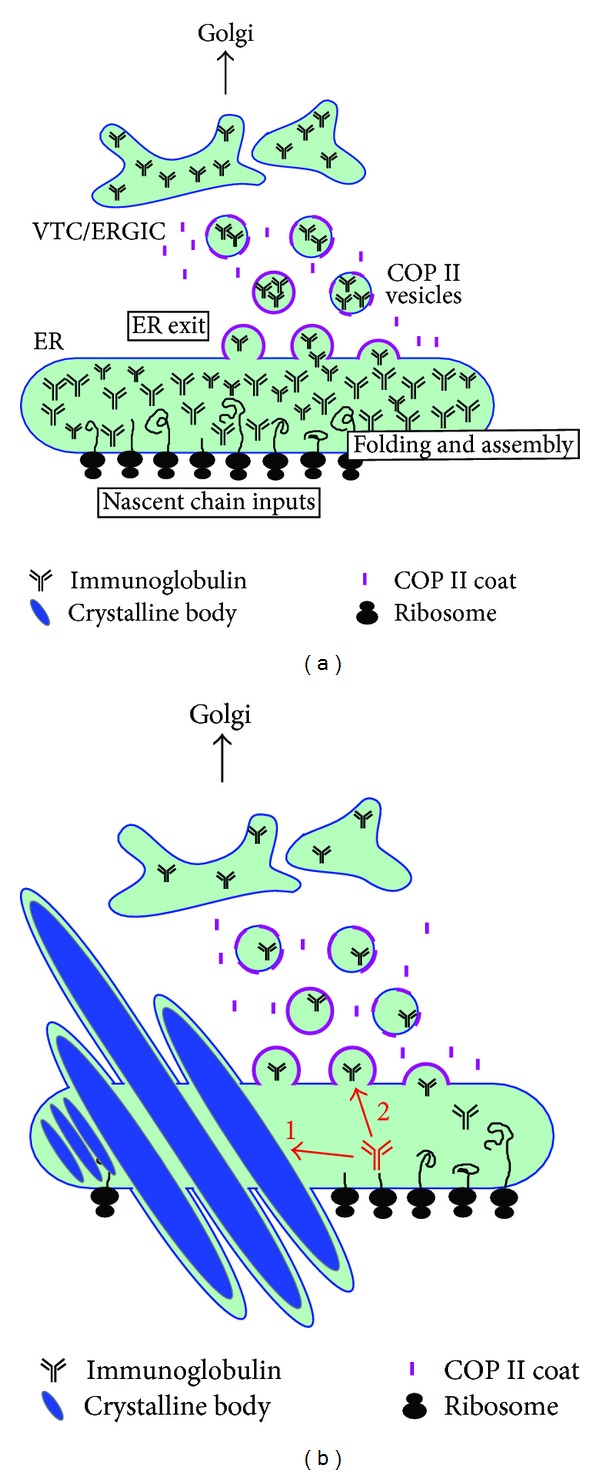
Schematics of early secretory pathway compartments during recombinant IgG overexpression. (a) Increased efficiency in protein synthesis, translocation, and oxidative protein folding, in conjunction with limited capacities at the ER exit sites, can lead to a progressive accumulation of export-ready IgG in the ER lumen. VTC: vesicular tubular cluster. ERGIC: ER-Golgi intermediate compartment. (b) Upon reaching a critical threshold concentration, IgG crystals can form in the ER. Accumulated IgGs support the rapid growth of intra-ER crystals. Crystalline body formation in turn alleviates the crowding in the ER. Newly folded IgG will have two competing fates once the crystalline body phenotype is initiated. The first path is to become part of the growing crystals. The second path is to be packaged into COP II vesicles to exit the ER. After crystalline body is induced, many attempts to increase protein synthesis may not directly translate into a higher protein secretion output due to the two competing pathways.

Immunofluorescent imaging revealed that this model IgG clone tended to accumulate in the ER at steady state. Potential secretory bottlenecks, or the rate limiting trafficking steps, for this IgG clone were thus located at the pre-Golgi stage. Importantly, the cells that had yet to form crystals showed qualitatively much higher intra-ER IgG concentration compared to the cells already housed crystals [102]. These observations suggested a scenario in which the intra-ER IgG concentration reaches the highest level just before the crystal nucleation event, which is rate-limited by a certain threshold concentration. Once the crystals nucleated and started growing, molecular crowding in the ER was rapidly alleviated because preaccumulated IgGs supported the crystal growth quickly [102]. This model obviously predicts that the intra-ER crystal growth competes with the ER export machinery toward available pools of newly folded IgG species, as illustrated in Figure 3.

Because the pH of the ER lumen is reported to be ~7.2 in various cell types [103, 104] and the calculated isoelectric point of the model IgG was in the neutral pH range, it was suspected that the model IgG had low solubility in a neutral pH environment. In fact, when a concentrated IgG was formulated in neutral pH buffers, the IgG crystallized readily *in vitro* [102]. Thus, the crystallizing propensity was an intrinsic property of this model IgG clone in a neutral pH environment. Structural modeling revealed the presence of an acidic patch on the surface of the variable domain which was prominent, if not unique, characteristics of this IgG clone. The acidic cluster was composed of five aspartic acid residues in the VH CDRs and the neutralization of this negative charge patch by mutagenesis abrogated its crystallization propensity in the neutral pH solution *in vitro *[102].

The key piece of evidence toward the importance of variable domain properties in CB formation came from transient expression experiments using HEK 293 cells. When the parental IgG clone or the two acidic patch-neutralized mutants were transiently expressed in HEK 293 cells, none of the three tested IgGs spontaneously induced CB formation at steady state, and they were secreted equivalently. However, when the cells were treated with Brefeldin A to accumulate the export-ready IgG pools in the ER, only the parental IgG clone induced CB formation in nearly 30% of the transfected cells whereas the two charge-neutralized mutants did not [102]. The acidic patch on the HC CDRs hence played roles in the crystallization events both *in vitro* and *in vivo*. Importantly, the results also demonstrated that even when an IgG had a predisposed crystallizing propensity *a priori*, intra-ER crystal nucleation would not take place unless export-ready IgG accumulated above a critical threshold concentration. In some cells with high protein synthesis and folding capacities, the critical threshold concentration can be reached spontaneously. In contrast, some other cells required a blockade of ER-to-Golgi transport steps to accumulate export-ready IgG above threshold. Both intrinsic and extrinsic factors therefore played roles in CB formation, similar to the cases of RB formation. Hasegawa et al. [102] proposed that the striking cellular phenotypes of CBs can be induced when the efficiency of IgG protein synthesis and oxidative folding exceeds the capacity of the ER exit site functions. As a consequence, otherwise export-ready IgG progressively accumulates in the lumen until a critical concentration is reached. The threshold concentration can vary from clone to clone depending on the properties encoded in the variable domain sequences. Furthermore, the phenotype also suggested an interesting possibility that the physicochemical properties of the Igs themselves can directly influence the maximum secretory outputs from the cells expressing the Ig clone.

### 3.3. Implications of the Crystalline Body Formation for the Therapeutic IgG Screening and Expression Strategy

Unlike the numerous examples of unfolded protein accumulation in the ER that triggers severe ER stress responses [105, 106], accumulation of correctly folded proteins in the ER has not been well studied in animal cells. It is well known, however, cereal plants use the ER to store nutritionally important protein, during seed maturation, in the form of large ordered protein aggregates called protein bodies [107, 108]. In the recombinant CHO cells overexpressing the CB-inducing human IgG, all three arms of UPR signaling pathways appeared to be constitutively active [102]. Nonetheless, the IgG synthesis continued and newly folded IgGs were fed into the growing crystals without dying out of apoptosis, although the cells eventually die out of physical membrane puncture [102]. Similar to protein bodies in cereal plants, the CHO cells that develop intra-ER IgG crystals somehow effectively manage ER stresses during the crystal nucleation and elongation. Diverse roles of UPR signaling outside the realm of ER stress and protein misfolding have been accumulating (reviewed in [109]); however, the mechanisms of apoptosis resistance amid chronic UPR activation are still elusive for CB-housing cells. Clarification of such mechanisms may benefit cell engineering approaches to develop highly productive recombinant cell lines for biotherapeutic manufacturing. Furthermore, a detailed molecular understanding of the intracellular trafficking bottlenecks at the ER exit sites that apparently played roles in CB phenotype may also provide new insights into cell engineering strategies to increase cell's secretory capacity further.

Because intra-ER IgG crystal formation can compete with the ER export machinery to gain access to the new supply of properly folded, export-ready IgG species, selecting such clone as a lead candidate can come with a cost (Figure 3). It is quite likely that the rational efforts to increase protein expression levels and to optimize cell culture conditions may not translate into increased secretion titers because the limit is most likely set by the concentration-dependent crystal nucleation event in the ER as described in Section 3.2. The IgG species ended up forming CBs are, in essence, “excess inventory” that could have been secreted if there were enough capacity at the ER exit sites. Unless there is an effective cell phenotype engineering strategy to enhance ER exit site capacity and function, selecting the IgG clones with high CB-inducing propensity can discount the benefit of all the available bioprocessing technologies. Those technologies may simply help cells reach the threshold concentration faster and initiate the phenotype cycle prematurely. The appearance of CB phenotype may additionally offer a convenient assay to detect low solubility issues in a neutral pH environment of the ER lumen that might preemptively report a potential issue in product formulation steps or even in *in vivo* settings at the physiological pH. Because there is only a limited set of examples where CB formation can be experimentally inducible, it is still largely untested whether the mechanisms proposed by Hasegawa et al. [102] would apply widely. Similarly, the predictive value of CB phenotypes in forecasting the aberrant solution behaviors is currently unknown.

### 3.4. Lysosomal Storage of Immunoglobulin Crystals

Although the primary site of CB formation is the ER of the Ig expressing cells, Ig crystals can also develop in the endo/lysosomal compartments of various target cells. A simultaneous formation of crystalline deposits both in the plasma cells (the primary site) and in the epithelial cells of renal tubules (the secondary site) has been reported in myeloma patients [110, 111]. Because plasma cells first secrete Igs that are then phagocytosed by the target cells away from the site of biosynthesis, the accumulation of protein crystals in the afflicted target cells can be considered as a distal event [83]. Because the lumenal pH of the ER and the lysosomes is distinct, and the former compartment is for protein synthesis while the latter is for degradation, the mechanisms of crystalline formation can be quite different in two separate subcellular locations.

#### 3.4.1. Adult Fanconi Syndrome

Crystal inclusions found in proximal renal tubule cells of myeloma-associated Fanconi syndrome patients are localized in the organelle of endosome/lysosome system. Interestingly, the crystals are always composed of free *κ*LC [112, 113] except for a few rare reported cases of free *λ*LC crystallization [114, 115]. Sequence analysis on Fanconi *κ*LCs revealed that the majority of them belonged to V*κ*1 variability subgroup and originated from two germline genes, O2/O12 and O8/O18 [113, 116, 117]. Particularly, the patients with O2/O12-derived sequence had a hydrophobic residue (position 30) exposed to the solvent in the CDR-L1 loop [113, 118] which confer apparent protease-resistance to a lysosomal enzyme cathepsin B [119, 120]. The current model postulates that physicochemical properties of the somatically mutated V*κ* sequences make these free LCs resistant to proteolysis and hindering normal catabolism. This is followed by the accumulation of predisposed LCs in the endocytic compartment of proximal tubule cells, eventually leading to intracellular crystallization [113]. The crystallized LCs evidently interfere with normal absorptive functions at the apical membrane and impair renal functions.

#### 3.4.2. Crystal-Storing Histiocytosis

Crystal-storing histiocytosis (CSH) is another example where Igs crystallize in the lysosomes of histiocytes, or macrophages, in the bone marrow, spleen, liver, lymph nodes, lung, and other organs [121–123]. Although CSH appears to develop in patients with plasma cell tumors expressing any HC classes, the involved LCs are almost always the *κ* isotype [111] and have the highest homology with V*κ*1 variability subgroup, germline gene O8/O18 [124, 125]. It is not yet fully clear whether the preexisting Ig crystals in the extracellular space were phagocytosed by macrophages and were retained in the endosomal organelles or whether the culprit Ig clones accumulated in the endocytic organelles before crystal nucleation took place *de novo* in this acidic compartment. Because some Igs are known to crystallize readily at an acidic pH at 37°C [126], there is a possibility of such low solubility Igs come to crystallize in the lysosomal lumen after taken up by the macrophages [127]. At the molecular level, unusual amino acid substitutions, including M4L in framework 1 and P59L in framework 3 regions within the O8/O18 germline gene sequence, were proposed to play roles in altering conformation, solubility, or susceptibility to lysosomal proteases [124].

Macrophages do express neonatal Fc receptor (FcRn) and actively recycle endocytosed IgGs back to the extracellular space, and thereby contributes to the IgG homeostasis *in vivo* [128, 129]. However, depending on the properties within the variable domain sequences, not all IgGs interact with FcRn equivalently and are salvaged from the degradatory pathway in an equally efficient manner, and it often results in significantly different serum half-life among different IgG clones [130]. It is attractive to speculate that, because of the properties embedded in the variable domain sequences, some IgG clones (particularly the ones with proteolysis-resistant mutations) preferentially escape salvage mechanisms and accumulate in the endo/lysosome compartments.

#### 3.4.3. Implications of Intra-endosome Crystallization in Recombinant Antibody Manufacturing

The mechanisms of intra-endo/lysosomal Ig crystallization in general are still unclear and such crystallization events have not been experimentally induced *in vitro* using commonly used heterologous production cell hosts such as CHO or HEK 293. Similar to any other soluble compounds in the culture media, secreted IgGs can be actively endocytosed by the cells residing in the same cell culture vessel during a protein production run by a process of constitutive fluid phase internalization. If the IgG clones happen to have a propensity to crystallize at an acidic pH and are resistant proteolysis, such IgGs may crystallize in the acidic compartments of the IgG expressing cells if enough high concentration is reached. The crystallization event in the endosomes can pose challenges during manufacturing in mammalian cell system, because the obstruction of endosomal trafficking or the mechanical damages to the membranes can compromise cell health, which in turn leads to a lower secretion titer.

## 4. Roles of Differential Physicochemical Properties in the Extracellular Fate of Immunoglobulins

Physicochemical properties of individual Igs will inevitably influence the fate of individual Ig clones differently in the extracellular space upon secretion. Extracellular fate such as serum half-life may often depend on whether the antibodies have bound to membrane-anchored cell surface target antigens (i.e., target-mediated clearance), yet other aspects may be independent of interactions with specific antigens or Fc receptors.

### 4.1. Amyloidogenic Immunoglobulins: Light-Chain Amyloidosis and Heavy-Chain Amyloidosis

Amyloidosis is a disease of protein misfolding where soluble secreted proteins aggregate into insoluble fibrils which are then deposited to tissues to cause functional and structural organ damage [131, 132]. In the LC amyloidosis, the N-terminal fragment of LCs composed of the variable domain and a part of constant region are reported to form organized fibrils. The LC amyloid can be stained by Congo red and it exhibits green birefringence in polarized light [133]. Unlike other LC-associated diseases, the *λ* isotype is more prevalent than *κ*, roughly by a ratio of 3 : 1 [134]. Among the *λ* isotypes, V*λ*3r and V*λ*6a variability subgroups are overrepresented in the tissue deposit [135, 136]. When it comes to the *κ* isotype, V*κ*1 and V*κ*4 variability subgroups are ascribed to be more amyloidogenic [137, 138]. Furthermore, depending on the variable region gene usage, different organs are differentially targeted. For example, the V*λ*6 was associated with renal amyloidosis, while the V*λ*2 and V*λ*3 were deposited in cardiac and soft tissues, respectively [137, 139]. V*κ* clones, in contrast, appeared to have affinity to hepatic tissues [137]. These results implicated the important roles of variable domain properties in organ tropism and even clinical outcomes. By analyzing 180 human monoclonal LCs, Stevens et al. identified a set of residue positions that may render some LCs amyloidogenic. For example, Asp at 31 in CDR1 of *κ*LC was a result of somatic mutation and showed high association with amyloidosis [140]. Similarly, Asp at position 50 located in the CDR2 of *κ*LC (V*κ*1) was encoded by O18/O8 and L18 germline gene segments and was prevalent in amyloidogenic *κ*LCs [140]. Their molecular modeling suggested that when the two VL domain dimers are stacked on each other, oppositely charged side chains (i.e., Lys-42 and Asp-50) are able to form salt bridges. This electrostatic interaction between variable domain dimers was proposed to initiate a slow, nucleation-dependent process to form fibrils [140, 141]. Furthermore, the study on V*λ*6 subgroup showed the presence of a salt bridge between Asp-29 and Arg-68 that protects LCs from fibril formation [142]. In addition, Helms and Wetzel [143] showed that Arg-61 and Asp-82 of *κ*VL domain form a conserved salt bridge between the two adjacent loops. In fact, a known amyloidogenic R61N point mutation destabilized the domain, and made it susceptible to induce fibril-like protein aggregation *in vitro* [143]. Likewise, D82L mutation was reported in an amyloidogenic Bence Jones protein [144]. A variant of this mutation (D82I) is also associated with LC deposition disease (Section 4.2, below) and can lead to amorphous aggregate formation *in vitro* [143]. This salt bridge therefore plays key roles in various LC disease phenotypes.

In spite of all the identified differences between amyloidogenic and nonamyloidogenic LC primary sequences, there was no single amino acid substitution or combination that was directly linked to fibril forming propensity. Instead of a particular primary sequence signature, a reduced VL domain folding stability was proposed as a unifying property of amyloidogenic LCs. If the LCs have a certain predisposition to assume a partially unfolded state due to amino acid substitutions, such LCs tend to be more amyloidogenic [145–147]. In fact, many known pathogenic amino acid substitutions were shown to destabilize the LCs and consequently increased the propensity to form fibrils [148]. It was also found that even a nonamyloidogenic LC can be induced to form amyloid fibrils if maintained in a mildly denaturing condition which increases the concentration of folding intermediate species [148, 149]. Therefore, any LCs can potentially aggregate into amyloids when somehow being stabilized in a state of folding intermediate [149].

There are rare clinical instances where the amyloid tissue deposits are composed of truncated forms or sometimes a fragment of Ig HCs and they are called HC-associated amyloidosis. In the very first reported clinical case, the HCs had an extensive internal deletions of CH1, hinge, and CH2; therefore, the amyloid protein was composed of the complete VH domain directly joined to the CH3 domain [150]. In the second case of HC amyloidosis, the renal and splenic amyloid deposits were made of the VH-D-encoded portion of the HC fragment—without any regions of constant domain [151]. Although these reports implicated the importance of variable domain properties, the understanding of the mechanisms is still insufficient.

### 4.2. Monoclonal Immunoglobulin Deposition Disease

Non-amyloid deposition diseases are categorized into three groups based on the components of amorphous Ig tissue deposits: light-chain deposition disease (LCDD), heavy-chain deposition disease (HCDD), and light- and heavy-chain deposition disease (LHCDD) [152]. Unlike amyloids, deposits are Congo red negative and amorphous and are nonorganized by electron microscopy [153].

For the LCDD, the involved LCs are predominantly *κ* isoform [154] and the V*κ*4 subgroup (B3 gene) is overrepresented [155]. Pathologic *κ*LCs typically have multiple amino acid substitutions [156, 157]. For instance, somatic mutations that resulted in the introduction of an N-linked glycosylation site in the variable domain has been reported, for example, D70N substitution (framework 3 region) in V*κ*4 subgroup [158] and T74N in V*κ*3 subgroup [159]. Other identified amino acid substitutions include the replacement of solvent exposed polar residues with hydrophobic ones in V*κ*1 subgroup [160]. Similarly, comparison of LC primary sequence revealed that the common features of pathologic LCs were the solvent exposure of hydrophobic residues in CDR1 and CDR3 regions also in the V*κ*4 subgroup [161]. These findings prompted a model that a clustering of hydrophobic residues creates a hydrophobic zone which favors enhanced LC aggregation and amorphous deposition.

In rare cases, amorphous deposits are composed of abnormally truncated HCs often missing the CH1 domain [162] or missing part of the variable domain in addition to the CH1 domain [55, 163]. As summarized in Section 2.3, the deletion of CH1 domain naturally takes place by imprecise class switching recombination events and this deletion facilitates the escape of HCs without assembling with LCs. Although the CH1 domain deletion seems to be required for an efficient extracellular release of HC-only protein, this structural abnormality was not sufficient for tissue deposition. In fact, similar to many other Ig diseases, the properties of the variable domain play important roles in HCDD [164]. For instance, various amino acid substitutions are often found in the framework regions that would change the physicochemical properties (i.e., charge and hydrophobicity) of VH domains [165]. There are isolated cases where both HC and LC comprise tissue deposits [166–168], but the molecular basis has not been well characterized [169].

### 4.3. Cryoglobulins

Cryoglobulins are a group of Igs that precipitate or become gelatinous upon cooling and become reversibly soluble upon rewarming to 37°C [170]. In rare cases, a higher temperature (e.g., 54–56°C) is required to become fully soluble again [171, 172]. The presence of some cryoprecipitating proteins in the serum had been known since the study on a multiple myeloma patient by Wintrobe and Buell [173], but the first detailed characterization on cryoglobulins was not reported until Lerner et al. [174]. While most proteins are generally soluble at 4°C even at concentrations above 100 g/L, some cryoglobulins are known to precipitate even at 1 mg/L [175–177]. According to Cream [178], cryoglobulins are present in normal serum sometimes up to 80 mg/L (on average ~30 mg/L). If present at high concentration, cryoglobulins can cause hyperviscosity at low temperatures that decrease blood flow and sometimes result in capillary occlusion or vascular damage [176, 179] or renal dysfunction [180].

Extracellular behaviors of individual cryoglobulins clones can vary widely. For instance, optimum pH range for cryoprecipitation, inhibitory pH range, critical concentration, temperature threshold, and the kinetics of precipitation can all vary from clone to clone even among cryoglobulins [177, 181–185]. There is one cryo-IgG clone that was shown to crystallize even at 37°C when the pH was shifted to a range between 5.0 and 6.5 [126]. The morphology and fine structures of individual cryoprecipitates vary from clone to clone considerably from crystalline to amorphous or gel, thereby further reflecting the physicochemical diversity of Igs that display cryoprecipitation phenotypes [186, 187]. Studies in 1960–70s were largely focused on the attempts to differentiate cryoglobulins from noncryos by amino acid contents [188–190] and various physicochemical studies were carried out [181], but the precise mechanisms of cryoprecipitation remained elusive.

Because the IgG3 isotype is often found in the cryoglobulins in both mouse and human [191, 192], more positively charged CH2 domain of the *γ*3 chain has been postulated in electrostatic interactions that lead to cryoprecipitation. Importantly, given that not all monoclonal IgG3s show cryoprecipitation, the properties of the variable domains inevitably come into play [193]. In fact, residues 6 and 23 of the VH domain appeared to be more positively charged in IgG3 cryoglobulins compared to noncryo-IgG3 [192]. Kuroda et al. [194] reported that cryogenic IgG3 in general had lower contents of terminally sialylated glycans than non-cryo IgG3. Even within the same cryogenic IgG3 clone, the species found in the cryoprecipitates had lower sialic acid contents than the species that still remained soluble in a same test tube. It was proposed that negatively charged sialic acids at the oligosaccharide terminus have inhibitory effect on IgG3 cryoprecipitation, by neutralizing the positively charged *γ*3 CH2 domain. In some cryoglobulins on the other hand, the Fc region seems to play less critical roles since the F(ab′)_2_ domain retained such propensity by itself for IgA [195] and IgG [196]. In the case of two cryo-IgG2 clones, both F(ab′)_2_ and Fab fragment retained cryogel forming propensity, although it required a higher concentration than the parental whole IgG molecules [197].

Since the surface of each Ig clone's variable domain has a unique distribution of amino acids, different types of non-covalent molecular interactions (e.g., hydrophobic, electrostatic, and hydrogen bonding) can take place between each other and with the solvent. It is believed that cryoglobulins in general have unfavorable attributes that hamper efficient interactions between the cryoglobulins and the solvents at low temperatures [198, 199]. Andersen et al. [199] proposed that temperature-dependent intramolecular conformational changes influence some Igs' solubility at low temperatures. Intramolecular hydrophobic bonds, for example, might be weakened at low temperatures with a subsequent unfolding of the molecule and exposure of normally masked residues for new intermolecular interactions. Changes in sedimentation behavior and viscosity as a function of temperature appeared to underpin the conformational changes at low temperatures for some cryoglobulin clones [200]. For a different set of cryoglobulins, however, such cold-induced intramolecular conformation change was not detected [181, 201]. An alternatively proposed model was the cooperative intermolecular association via a rate-limiting nucleation event that takes place above a critical concentration [201]. This model was supported by additional studies from another group using two independent cryo-IgG clones. Vialtel et al. [197] demonstrated that polymerization of the model cryo-IgG clones was a nucleation-controlled process that was rate limited by the initial thermodynamically unfavorable dimer formation. This polymerization process required a critical monomer threshold concentration above 2-3 mg/mL and the presence of a dimer as a stable intermediate (acting as a protomer) that primes the growth steps. Interestingly, only the dimer of autologous cryo-IgG can induce the polymerization reaction, and the dimers from a different cryo-IgG clone cannot prime this reaction, thereby revealing a direct role of variable domain sequence and its physicochemical property in cryoprecipitation reaction [197].

### 4.4. Cryocrystalglobulinaemia and Extracellular Immunoglobulin Crystals

In cryocrystalglobulinemia [202], not only can the culprit monoclonal Igs crystallize in the cytoplasm of plasma cells that express them, but the secreted counterpart of the same Ig clone also deposits as extracellular crystals, thereby believed to cause microvasculature lesions and to impair renal functions [203, 204]. Rengers et al. [205] identified a mouse monoclonal IgG3 clone 8A4 (raised against hydrogenase from *Wolinella succinogenes*) that spontaneously formed intracellular crystals in the hybridoma cells during a normal cell culture condition at 37°C. The secreted version of IgG3 clone 8A4 simultaneously exhibited cryoprecipitating properties *in vitro*. This observation illustrated that some overlapping physicochemical properties can be responsible for both CB formation and cryoprecipitation. Using this model clone, Rengers et al. [205] demonstrated that the variable and the constant domains of both subunits are collectively responsible for this unique phenotypic outcome. For instance, the 3-amino acid deletion (-S-V-E-) in the mouse V*κ* CDR1 region not only increased the secretion level 7-fold, but also abrogated its intracellular crystallization propensity within the hybridoma cells. Furthermore, class switching to IgG1, with no change to the variable domain, prevented the crystal formation, showing the importance of the interaction between the variable and the constant domain in this particular crystallization event—or alternatively, the constant domain isotype simply modulated the crystallization threshold. When the 8A4 hybridoma was grafted to mice, the hybridoma formed tumors composed of crystal-laden cells. Moreover, antibodies secreted from the established tumors induced renal damage by forming crystals along the glomerular basement membrane. In contrast, when the hybridoma that secretes SVE-deletion variant was grafted, the secreted IgG3 caused renal legions but by forming amorphous deposits instead of crystals. Physicochemical properties of IgG clone therefore not only can change the pattern of extracellular deposit, but also can change the type of nephrotoxicity *in vivo* [205].

### 4.5. Pyroglobulins

Pyroglobulins are Igs that become insoluble, sometimes even become a firm gel, in a temperature range of 56–60°C [206]. Unlike cryoglobulins, however, returning the temperature to 37°C does not reverse their insolubility. The importance of variable domains is implicated by the representation of all Ig isotypes in the family of pyroglobulins—IgD/*λ* [207], IgM/*λ* [208], IgGs [209], IgA/*κ* [210], and IgE/*κ* [211]. Because its characteristic insolubility occurs at temperatures far above a normal physiologic temperature range, thermoprecipitation of pyroglobulins is not expected to cause clinical symptoms readily even if they are produced at high level *in vivo*.

Depending on the physicochemical properties of Igs as well as the mode of intermolecular interactions, the thermoprecipitability can be abolished by treating with different denaturants (SDS, urea, or guanidine-HCl), neutral salts (NaCl or MgCl_2_), reducing agents, sugars, pH, or ionic strength. However, not all the treatments work equally well in preventing different pyroglobulin clones from forming gels. Therefore the mechanism and biochemical basis of pyrogel formation appears to be quite diverse from clone to clone. Interestingly, irrespective of whether the intermolecular disulfides are intact or not, the presence of both HC and LC appears to be required for some cases [207].

Studies carried out in the 1970s largely focused on determining amino acid compositions of pyroglobulins and comparing against normal counterparts [212, 213]. Although this type of analysis on a small sample number did not yield helpful insights into the mechanism of thermoprecipitation, the rationale was to uncover the possible difference in physicochemical properties, if any, between the group of pyroglobulins and a subset of normal Igs. Nevertheless, the recurring models of those studies were twofold. (1) Somatically introduced mutations to the VH and VL domains may lead to abnormal conformation or loss of surface polarity so that solubility can be maintained only by forming aggregates at a physiologic temperature. (2) Temperature elevation may cause conformational changes that expose additional nonpolar residues of the hydrophobic core for new intermolecular interactions, followed by an irreversible hydrophobic interaction and gel formation at 56°C [177, 212, 213]. Depending on the properties of VH and VL, perhaps in conjunction with constant domain sequences, some Ig clones do possess combined properties of both cryoglobulins and pyroglobulins at different temperature ranges [214, 215]. This observation highlights further that the same set of physicochemical properties could manifest as different phenotypes under different environment.

## 5. Synthesis of Engineered IgGs and Antibody Fragments

Applications of histidine-based pH-sensitive target binding functions in therapeutic antibodies have gained popularity in recent years [216, 217]. In this group of engineered IgGs, antibodies are intended to bind to its target at high affinity in the extracellular space (pH ~7.4), but lose its affinity to the same antigen in the acidic pH of endosomal compartments. This mode of biphasic binding affinity is expected to facilitate a recycling of the “used” antibodies back to circulation via FcRn-mediated pathway for a fresh round of antigen capture, while, in contrast, the target antigen is sorted to lysosomes for degradation without getting recycled. These antibodies can escape target-mediated clearance mechanisms (particularly when the target antigens are membrane-anchored cell surface proteins) and are expected to show longer serum half-life than those which do not have such properties. Because of their mode of action, it is possible that their solution behavior can be more complex than the “normal” Ig counterparts depending on the protonation state of the purposefully placed histidine residues. Moreover, similar to the endosomal trafficking system, compartmental pH gradients also exist in the secretory pathway. In fact, by taking advantage of a pH gradient from the ER to the Golgi compartment, the functions of some resident proteins are intricately tied to the compartmental pH difference [218–220]. Detailed studies on the biosynthesis of pH-sensitive antibodies have not been reported to date, but the group of histidine-engineered IgGs may turn out to serve as a unique tool for cell biologists to probe unexplored functions of the secretory pathway organelles.

Because of the apparent modular nature of Ig structure, a number of alternative antibody formats have been explored and proposed as potential therapeutics [221–223]. Despite various design possibilities, the most critical components of those antibody-based alternatives are nevertheless the VH and VL sequences. In some designs, the molecules are composed of VH, VL, or scFv as a building block to achieve dual- or multiantigen specificity. Because of their structural formats, the physicochemical properties of the variable domains may influence the biosynthetic steps and the solution behavior more directly than those of conventional IgG-like format. An extensive study on the thermostability and the production yield of scFv fragments (using a systematic panel of VH and VL germline family consensus domain combination) implicated that small number of VH/VL pairs (e.g., VH3/V*κ*3, VH1b/V*κ*3, or VH3/V*κ*1) possess superior biophysical properties [224]. Therefore, careful selection of VH/VL sequence pairs may become even more critical to ensure high biosynthetic efficiency and compatibility as well as to rectify their behaviors in the extracellular space. Given that different HC isotypes with identical variable domain sequence alone can alter the threshold to induce RB formation (Section 2.4), it is likely that a molecular format of alternative antibody fragments has pronounced impacts on the RB-inducing threshold. However, the precise relationships between the molecular format and the biosynthetic load to the cell host are not well understood. Similarly, given that the differences in the HC constant domain isotype alone can impact the physical nature of paratopes and consequently alter how the antibodies interact with the cognate target (e.g., fine specificity, affinity, kinetics, functionality) [225–228], it can be envisioned that alternative structural format can unpredictably influence the nature of their antigen binding. Furthermore, it has been shown that despite sharing the same IgG isotype, the clonal differences in VH/VL sequence alone have considerable impact on how IgGs bind to FcRn and on the pharmacokinetics of individual IgG clones *in vivo* [130, 229]. Clearly, the physicochemical properties of Fab and Fc domains are not entirely independent of each other although the modular configuration of Ig structure may suggest as if they are. Those observations may pose interesting challenges for the alternative antibody fragment designs to achieve desired antigen binding properties and serum half-life.

## 6. Conclusion and Prospective

Nearly every aspect of Ig structure and function can be engineered to improve selected properties to a desired level, to confer additional effector functions, or even to eliminate undesirable attributes. A number of research articles and reviews are already available on such subjects [230, 231]. Instead of reiterating the examples of protein engineering success, this paper has employed physicochemical properties as guiding themes to illustrate their influence on the fate of Igs during biosynthesis and upon secretion. Numerous clinical cases suggested that some germline gene segments are apparently more prone to generate pathogenic polypeptide sequences than others. Likewise, somatically introduced amino acid substitutions contribute to create physicochemical properties that underscore peculiar behaviors of some Ig clones. Furthermore, structural abnormalities caused by truncation or internal deletions also modulate the threshold for certain biochemical and biophysical propensities. Regardless of engineered or not, individual Igs possess unique physicochemical properties embedded in its variable domain sequences, sometimes in conjunction with different HC isotypes.

Instead of regarding the cellular system as a black box, it is undoubtedly important to pay close attention to the fate of secretory cargo inside of the cells, particularly in the ER—because this is where secretory proteins come into being. Studies on the biosynthesis and intracellular trafficking of antibody therapeutics are rather limited as if to show that there is little to learn or as if all the recombinant IgGs behave similarly, if not identically, when they are still inside of the cells—or as if individual IgG clones start revealing their unique characters only after they are purified to homogeneity and bottled in glass vials to run various *in vitro *stress tests. Unless there are primary sequence-based reliable prediction methods, RB and CB formation can be one of the earliest observable phenomena that report us valuable information on how the host cellular systems interact with heterologous secretory cargoes, although it took us roughly 100 years before we recognized their potential values in the biotechnology of recombinant IgG expression. In a way, we are able to see the live broadcasting of potential biosynthetic challenges while the events are actually unfolding in the *bona fide* biological systems, as opposed to the prediction algorithms.

Recombinant monoclonal antibodies that exhibit characteristics similar to the groups of Igs illustrated in this paper may not be suitable for therapeutic use for various reasons—in order to maximize the manufacturing success or to avoid the potential risk of vascular and renal damage. Because protein therapeutics are typically transported and stored in a refrigerated environment, some propensities, such as cryoprecipitation, may be more important than other attributes such as thermoprecipitation. Although this is beyond the scope of this paper, many of the unfavorable solution behaviors can be corrected by various pharmaceutical formulation strategies *in vitro*. However, it is important to keep in mind that once administered, biotherapeutics reside in the physiological environment where their characters can appear again even though they might have been suppressed by effective formulation methods. Because antibody biopharmaceutics are thoroughly purified and assessed for their structural integrity, there is little chance that administered IgGs can cause LC-associated diseases unless disulfide linkages become quickly labile in the circulation or during endosomal trafficking steps. Nonetheless, if the LC happens to belong to particular variability subgroups overrepresented in immunoglobulin diseases or have the same amino acid substitutions reported in well-characterized pathologic LCs, a precaution might be warranted. For the success of antibody therapeutic programs, it is therefore critical to identify lead candidates that not only possess desired therapeutic activities, but are also endorsed by biosynthesis-friendly physicochemical attributes that are underpinned by favorable VH and VL sequence pairs.

Hypothetically speaking, if we could line up 10^10^ individual Ig clones in one axis based on a specific attribute (e.g., viscosity, isoelectric point, or thermal stability, under a given condition), the distribution is most likely to be continuous from one end to the other end without a major gap. We can readily envision that there will be a continuum distribution in every measurable attribute we can think of. The recombinant protein technologies have allowed us to fine-tune many aspects of protein function and physicochemical properties by protein engineering—as long as they are measureable. However, many characteristics are likely to be interrelated and often interconnected in both explicit and tacit ways, so that an attempt to change one particular trait can result in altering other attributes that were not intended to be changed. Because we are inclined to measure only a particular set of parameters that we are interested in, we cannot fully know the true ramifications of a certain amino acid substitution on the parameters that we rationally decide not to measure. Besides, there may be some parameters and attributes that we still do not understand to be important or parameters too difficult to measure. There may even be parameters as yet undiscovered. With all the possible ranges of properties that can influence the fate of Igs from their synthesis to degradation, there are certain ranges of physicochemical “sweet spot” that we consider acceptable, or even favorable, for an ideal lead candidate—until we encounter the next Black Swan event [232] that gives us a new set of insights and framework of thinking.

Although the examples discussed in this paper may represent some of the extreme cases and perhaps rare occurrences, it is not difficult to envision that any IgG clone can show a modest level of undesirable attributes that can easily be overlooked unless they are disastrous. Therefore, it will continue to be a challenge for us to determine—of all physicochemical diversities possible—the range of attributes that are favorable, acceptable, and unacceptable as antibody therapeutics. With new alternative Ig-like protein modalities on the horizon, the technologies of recombinant Ig expression will continue to feed opportunities for cell biologists to investigate biological processes of secretory protein biosynthesis and secretory pathway trafficking as well as the protein recycling events in the endocytic pathway.

## Abbreviations

CB: Crystalline body
CDR: Complementarity-determining region
CHO: Chinese hamster ovary
ER: Endoplasmic reticulum
Fab: Fragment antigen-binding
Fc: Fragment crystallizable
HC: Heavy chain
HEK: Human embryonic kidney
Ig: Immunoglobulin
LC: Light chain
RB: Russell body
scFv: Single-chain variable region fragment
VH: Heavy chain variable domain
VL: Light chain variable domain.
